# Temporal Dynamics of Top Predators Interactions in the Barents Sea

**DOI:** 10.1371/journal.pone.0110933

**Published:** 2014-11-03

**Authors:** Joël M. Durant, Mette Skern-Mauritzen, Yuri V. Krasnov, Natalia G. Nikolaeva, Ulf Lindstrøm, Andrey Dolgov

**Affiliations:** 1 Centre for Ecological and Evolutionary Synthesis (CEES), Department of Biosciences, University of Oslo, Oslo, Norway; 2 Institute of Marine Research, Bergen, Norway; 3 Murmansk Marine Biological Institute, Murmansk, Russian Federation; 4 White Sea Biological Station, Department of Biology, Lomonosov Moscow State University, Moscow, Russian Federation; 5 Institute of Marine Research, Tromsø, Norway; 6 Knipovich Polar Research Institute of Marine Fisheries and Oceanography (PINRO), Murmansk, Russian Federation; Technical University of Denmark, Denmark

## Abstract

The Barents Sea system is often depicted as a simple food web in terms of number of dominant feeding links. The most conspicuous feeding link is between the Northeast Arctic cod *Gadus morhua*, the world's largest cod stock which is presently at a historical high level, and capelin *Mallotus villosus*. The system also holds diverse seabird and marine mammal communities. Previous diet studies may suggest that these top predators (cod, bird and sea mammals) compete for food particularly with respect to pelagic fish such as capelin and juvenile herring (*Clupea harengus*), and krill. In this paper we explored the diet of some Barents Sea top predators (cod, Black-legged kittiwake *Rissa tridactyla*, Common guillemot *Uria aalge*, and Minke whale *Balaenoptera acutorostrata*). We developed a GAM modelling approach to analyse the temporal variation diet composition within and between predators, to explore intra- and inter-specific interactions. The GAM models demonstrated that the seabird diet is temperature dependent while the diet of Minke whale and cod is prey dependent; Minke whale and cod diets depend on the abundance of herring and capelin, respectively. There was significant diet overlap between cod and Minke whale, and between kittiwake and guillemot. In general, the diet overlap between predators increased with changes in herring and krill abundances. The diet overlap models developed in this study may help to identify inter-specific interactions and their dynamics that potentially affect the stocks targeted by fisheries.

## Introduction

The Barents Sea is an open Arcto-boreal shelf-sea with an average depth of about 230 m. This ecosystem is both of large applied interest due to the large commercial fisheries, and also an interesting biological system showing clear bottom-up effects [1], [2], top-down effects [3]–[5] and climate effects [1], [6]. The climate appears to have a strong effect on the trophic control in the Barents Sea in that both climate and trophic control change with a decadal periodicity [7]. Understanding linkages between climate and trophic interactions is important for understanding the changes in the Barents Sea biodiversity expected to follow climate and harvesting changes.

Fairly simple pelagic Arctic ecosystems such as the Barents Sea [8] may be more vulnerable to changes in the abundance of the few key species [9] compared to more diverse system in terms of link strengths [10]. For instance, the collapse of the Barents Sea capelin *Mallotus villosus* stock in the 1980s significantly affected several trophic levels including the capelin prey, zooplankton [11], capelin predators such as the Northeast Arctic (NEA) cod *Gadus morhua* [12] and the harp seal *Pagophilus groenlandicus* [13], and alternative prey of capelin predators such as shrimp [14], [15].

In recent years, understanding and predicting food web dynamics in the Barents Sea have become a priority with the aim at improving the management of marine resources. As a result, there has been an increased focus on ecosystem or multispecies models [16]–[18]. Indeed, on an ecological time scale, top-predators can affect the abundance of other species through predation or competition. The top-predator (species at the top of their food chain) community in the Barents Sea consists of about 33 seabird species [19] and 21 mammal species [16], in addition to large demersal fish, of which cod is the most abundant species. Interactions between several of these species have previously been identified, such as between the Black-legged kittiwake *Rissa tridactyla* and the Common guillemot *Uria aalge* [20], between the Minke whale *Balaenoptera acutorostrata* and the NEA cod [21], and between the harp seal and the NEA cod [22]. Such interactions should be included when investigating population dynamics in an ecosystem context, i.e., taking into account species interactions.

The sensitivity of each top predator species to changes in prey availability depends on the availability of, and ability to use, alternative prey species. The sensitivity of the top predator community, being the sum of each predator sensitivity, depends on the response diversity within the community [23]. If the majority of the predators of the community responds to a stressor in the same way then the predator community will show a sensitivity to the change of this particular stressor. On the contrary, if the predator responses are diverse, the community is more robust. Based on the strong effects across species of the two first capelin collapses we may expect a low response diversity and hence a sensitive community. However, the top predators are typically generalists, foraging on different prey species depending on spatiotemporal overlap between predator and prey distributions, and abundance of the different prey species in the system [24], [25]. It is therefore likely that the response diversity of these top predators may depend on the availability of alternative prey in the system.

In this study we explore the interactions between some of the major top predators of the Barents Sea ecosystem, by investigating the diet overlap among them. These species may only interact (e.g., compete for food) if they share a certain amount of the prey resources. The prey availability in the system is varying both due to natural cycles [e.g., capelin, 26] and to anthropogenic pressures [e.g., fishing, 27]. We therefore expect that the top predator diets are varying through time. If the predators demonstrate species specific responses to changing prey abundances the number of response types may be high and lead to a year-to-year changes in both trophic and competitive interactions. To address these topics we have conducted a diet overlap analysis based on stomach content over the years and run generalised additive model to try to explain the temporal changes observed. We expect that the diet changes may be due to changes in prey abundance and distribution as well as in climate that can affect those.

## Methods

### Data

Following a simple food web description of the Barents Sea [8], the main predators in terms of total consumption [16] are the NEA cod (thereafter cod), the Minke whale, the harp seal and seabirds. The latter group includes black-legged kittiwake and the common guillemot, thereafter kittiwake and guillemot respectively. The diet data of four of these species is displayed in Table 1 and Fig. S1 in File S1; unfortunately the harp seal was omitted from the analysis due to sparse data.

**Table 1:** Species studied.

Species	Description and source	Years
Black-legged kittiwake *Rissa tridactyla*	Regurgitation of 653 adults on the breeding colony Kharlov Island on the coast of the Barents Sea (BS) during the breeding season (April-May).	1982–1999 (lacking data for 1984 and 1985)
Common guillemot *Uria aalge*	Observation of 1951 fish deliveries at the breeding colony Kharlov Island on the coast of the BS during the breeding season (April-May).	1984–1999 (lacking data for 1985)
NEA cod *Gadus morhua*	Stomach content a ^,^ b. To compare with the seabirds we used data for 68–72°N and 20–40°E only (Mar-July) and to compare with the whale data for 70–80°N and 5–40°E (July-Sept). c	1984–2009
Minke whale *Balaenoptera acutorostrata*	Stomach content of 345 whales caught in the BS between May-Sept. To compare with the seabirds a subset for the area <75°N was used. d	1992–2004

a Report of the ICES Arctic Fisheries Working Group [61], Table 1.3 p 55.

b The Russian-Norwegian data base on cod diet, further details see Mehl and Yaragina [31], and Dolgov et al. [30]

c the subsets from this base.

d Further details on the capture and the stomach sampling is given in Haug et al. [62]

### Collection of data, spatial and temporal extent of data

The origin of the diet data used is summarized in the Table 1. Data were transformed to annual average percentages by mass. To make inter-specific comparisons we merged some prey categories (see supplementary material, Table S1 and S2 in File S1). Seabird diets were obtained during the breeding period at Kharlov Island on the coast of the Barents Sea [28], [29]. We calculated the whale diet for the entire Barents Sea as well as for a subset of the data restricted to the southern Barents Sea (Minke whale sampled south of the 75°N, Fig. S1 in File S1). The complete data are used to analyse the change in the whale diet over time and to compare with the diet of the cod. The subset data are used to make inter-specific comparisons with seabirds because they are central place foragers and limited to the southern Barents Sea during breeding (the period when the seabird data were collected). Data on cod diet were taken from the joint Russian-Norwegian PINRO-IMR data base [30], [31], diet for the entire Barents Sea as well as for a subset of it (<72°N) to compare with seabirds were calculated.

We used two types of environmental variables, climate indices and prey abundances, as predictors in the statistical analyses (given in Table 2). As climate indices we considered the average Barents Sea surface temperature (ST, annual), an index of the areal coverage of cold, Arctic water in the Barents Sea and the winter North Atlantic Oscillation index (wNAO). The rationale for analysing the effect of climate indices on the diet changes is that these variables may influence the spatial distribution of both the predators and the prey [25], [32], [33], which is unknown in our study. Temperature influences zooplankton productivity [34] and also acts as a proxy for various direct and indirect effects [35]. In particular, high temperature has been associated with inflow of warm, and potentially zooplankton-rich, waters from the Norwegian Sea [35]. The North Atlantic Oscillation index measures large-scale climate effects, is positively correlated with inflow and temperature, and was found to be the best climatic predictor of zooplankton biomass in the Barents Sea in spring and summer [e.g., for plankton 5] and thus linked to the productivity of the system.

**Table 2:** Explanatory variables used for the GAM analyses. Subscript *t* refers to year.

Variable	Description and source
*ST _(t)_*	Mean Barents Sea (BS) temperature in °C for and January*_t_* to December*_t_* at 0–200 m depth in Atlantic water parts of the Kola section (70.5–72.5°N, 33.5°E) over 1921–2009^a^.
*NAO_(t)_*	Principal component based winter (December*_t-1_* – March*_t_*) North Atlantic Oscillation (NAO) index^b^
*Cap _(t)_*	Biomass of capelin in the BS in 10^3^ t^c^.
*Krill _(t)_*	Euphausiids, abundance indices covering 1984 to 2004 from the Polar Research Institute of Marine Fisheries and Oceanography (PINRO)^d^. Data are for southern (Krill.S) and the northwestern (Krill.NW) BS. Krill is the sum of both area.
*Herr _(t)_*	Biomass of immature Norwegian Spring Spawning herring (1–2 years of age) in the BS in 10^3^ t ^e^.

a Tereschenko [63, http://www.pinro.ru/], ^b^Hurrell [64, https://climatedataguide.ucar.edu/sites/default/files/climate_index_files/nao_station_djfm.txt], ^c^Report of the ICES Arctic Fisheries Working Group [65], Table 9.5 p 498, ^d^Zhukova et al., 2009 data used with permission, and ^e^Report of the ICES Arctic Fisheries Working Group AFWG Table 9.6 p 499.

Capelin, euphausiids (krill) and, to some extent also, juvenile Norwegian Spring Spawning herring *Clupea harengus* (thereafter herring) were found to be the major prey species in all predators (see Fig. S1 in File S1). These prey species are also considered major players in the trophic dynamics of the Barents Sea [e.g., 26, 35–39]. Thus the abundances of these prey species were used as predictors in our models (Table 2).

### Diet overlap Index and Niche breadth

Schoener's [40] index of niche overlap, which is the most commonly used diet overlap index [41], [42], was used to calculate the diet overlap among predators:
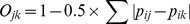
where *O*_jk_ is the overlap between the species j and the species k, p_ij_ is the proportion of species j feeding on prey species/group i and p_ik_ is the proportion of species k feeding on prey species/group i. *O*_jk_ values range from 0 to 1. Overlap in diet between species j and k is complete when *O*_jk_ = 1 and is absent when *O*_jk_ = 0 [20], [41]. Values exceeding 0.6 are considered to represent “biologically significant” overlap in diet composition [42]. However, we considered that when mean *O*_jk_>2˙SD the diet overlap between species j and species k is significant [20], [41].

Using original (non-merged) diet data, we have calculated the Schoener's index *O* for consecutive years (overlap of diet between years) for each predator species (kittiwake, guillemot, Minke whale, and cod). To do this we adapted the equation above to calculate diet overlap between years, replacing P_ij_ with P_i,j,t_ and P_i,k_ with P_i,j,t+1_, where j denotes a predator species and t year. This way we obtained a diet overlap *O*_t_ between year t and t-1 and ultimately a time series of *O* indices of temporal trends in diet overlap between years within the species. Furthermore, year to year changes in *O* were then related to environmental descriptors using Generalized Additive Model (GAM, see below).

We have also calculated *O* for each pair of predators over common period of time, and *O* for each pair of predators from year to year.

To assess the complexity of the diet for each species, we used the Shannon-Wiener niche breadth index D [43]. The D index has the advantage of not being greatly affected by sample size. D was calculated as follows: 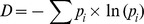
where p_i_ is the proportion of the species considered feeding on prey species/group i.

### Statistical analyses

The temporal variability in diet overlap (O_t_ ranging from 0 to 1) was analysed with respect to prey abundance and climate variables (both regional and large-scale climate indices) using Generalized Additive Models (GAM) with a logit link function in the formulation (family quasi-binomial) using the mgcv library in R 2.14.1 [44], [45]. Note that the quasi-binomial distribution takes into account overdispersion and underdispersion of the data.

We then modelled the observations Ot as coming from a quasi-binomial distribution with an expected value equal to logit(α+Σ_i_ s_i_ (X_i,t_)) where s_i_(˙) is a nonparametric smoothing function of covariate X_i_ on the dependent variable O. Note that the GAM analysis was conducted only for the predator pairs where the diet overlap *O* was considered significant (i.e., pairs where the diets overlapped over the whole studied period and not for some particular years only; see above).

The GAM procedure automatically selects the degree of smoothing based on the Generalized Cross Validation (GCV) score. GCV is a proxy for the model's predictive performance analogous to the Akaike's Information Criterion. However, to avoid spurious and ecologically implausible relationships, we constrained the model to be at maximum a quadratic relationship implying that we set the maximum degrees of freedom for each smooth term to 2 (i.e., k = 3 in the GAM formulation). The maximum number of explanatory variables on the starting models was depending on the time series length (number of variables should be ≤ to n/4, see Table 3). These explanatory variables were selected on two criteria that were availability and biological meaning.

**Table 3:** Results of the generalized additive models selected by shrinkage method of the relationship between diet overlap and different explanatory variables.

Overlap	Species	Variable 1		Variable 2		Variable 3		Variable 4		n	R^2^
**Intra-specific**	Kittiwake	ST _(t)_*	1.87	ln(Cap) _(t)_	1.59	Year _(t)_	0.00			14	0.66
	Guillemot	ST _(t)_**	1.98	Cap _(t)_	0.40	Year _(t)_**	1.00			14	0.78
	Minke whale	Herr _(t)_*	1.72	wNAO _(t)_*	1.01	Cap _(t)_	0.00			12	0.52
	Cod	ln(Cap) _(t)_*	1.92	Year _(t)_***	1.06	ST _(t)_	0.00	Krill_North__(t)_	0.00	23	0.55
**Inter-specific**	Minke whale vs cod	ln(Herr) _(t)_*	0.85	Krill _(t)_*	1.83	Year _(t)_	0.00			13	0.66
	Kittiwake vs guillemot	ln(Herr) _(t)_·	0.58	wNAO _(t)_*	1.79	Year _(t)_·	0.68			14	0.70

Models are written *O_t_* = α+*s_1_* (*X_1_,_t_*)+*s_2_* (*X_2_,_t_*)+*s_3_* (*X_3_,_t_*)+…+ε_t_, with ***s_i_***, a nonparametric smoothing function specifying the effect of the covariates *X_i_* on the dependent variable *O* for year t; **α**, intercept; and **ε**, stochastic noise term. The estimated degrees of freedom (edf) for each explanatory variable is indicated as is the significance (** *p*.Value <0.01, * <0.05 and **·** <0.10). Variables with edf = 0.00 were shrank by the fitting procedure and thus effectively removed from the formulation. See Fig 2–3 for the model fit and for the confidence intervals of the retained variables.

We wanted a parsimonious model which described the response well but was as simple as possible. We entered every candidate predictor in a GAM model and conducted a shrinkage model selection by using thin plate regression spline smoother with “shrinkage” for each term of the model [46]. Unimportant terms were shrank to zero, i.e., effectively removing the term, by the fitting procedure, and thus selecting a reasonably optimal model in one step (i.e. the model that includes all of the terms that were not shrunk to zero). There was no temporal autocorrelation (using autocorrelation function ACF) in the residuals of the models.

## Results

Capelin was the overall most important prey for the selected top predator species, ranging from an average of 27.5% in Minke whale diet to 34.9% in guillemot diet (Fig. S2 in File S1). However, sandeel was the most important prey for the guillemots (ca 49%) and krill was the most important prey for the Minke whale (ca 40%), when considering the whole Barents Sea. In these two cases capelin was the second most eaten prey. Herring was also an abundant prey in the diet of the predators (13–24%, apart for the cod where it represented only 2.7%) as was the krill (10–40%, apart for the guillemots which are not foraging on krill).

**Figure 1:**
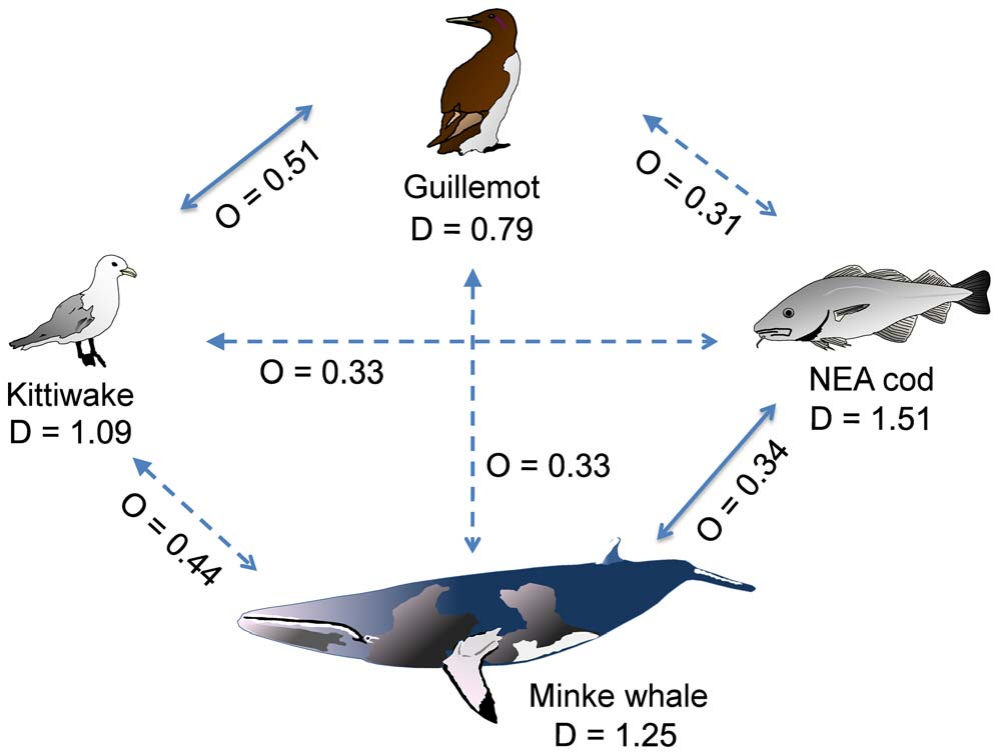
Trophic relationships between the main components of the food web in the Barents Sea ecosystem. Average Schoeners' diet overlap index *O* for the five predator pairs studied and their respective Shannon-Wiener niche breadth D (see Table S1 in File S1). The significant relationship are given in plain arrows (Fig. 3, Table 3) The shape of the arrow head indicates the interpretation on how one species may affect another based on biomass [55]. Different arrow heads indicate unbalanced biomass between a predator pair (filled head indicates a potential stronger effect than open head).

Diet breadth results show that the two central placed foragers (i.e., the two seabird species) had a narrower diet than the two other species (Minke whale and cod, Table S1 in File S1). Figure 1 gives the niche breadth for each predator. The cod had the broadest diet, followed by the minke whale, the kittiwake and the guillemot.

### Intraspecific year to year variation in diet

Table 3 shows the best models selected by shrinkage technique explaining the year to year change in diet for each predator.

**Figure 2:**
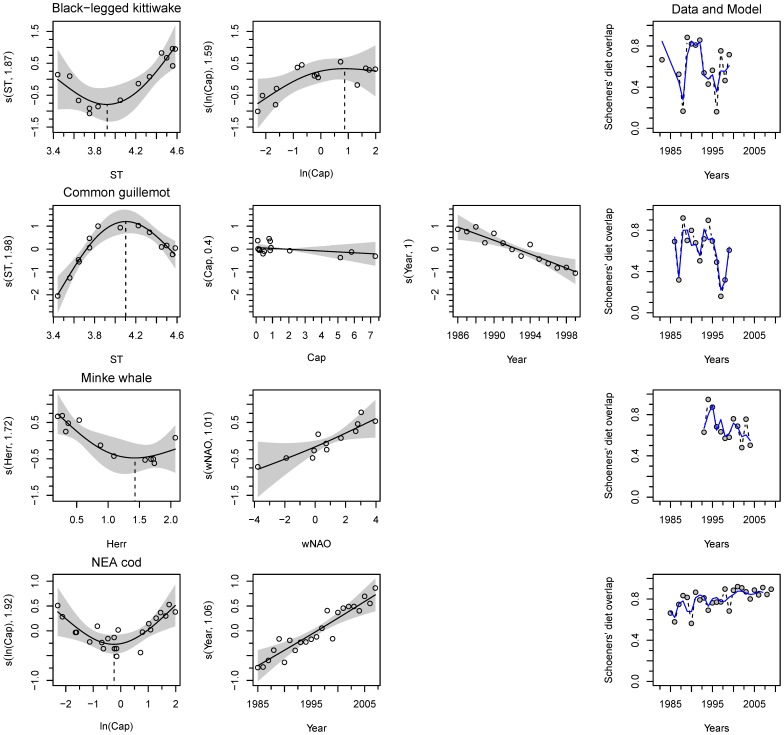
Intraspecific diet dynamics of the main predator species in the Barents Sea. The generalized additive models (GAMs) are presented for each predator. For each plot, the x-axes show the covariate and the y-axes the partial effect that each covariate has on the response variable. The line is the smooth term effect of the considered covariate on the elasticity with the pointwise 95% confidence interval around the mean prediction (grey-shaded area). The dots are the partial residuals calculated by adding to the effect of the concerned covariate to the residuals, the model prediction at any given point is given by the sum of all partial effects plus a constant. When it applies, the dotted line locates the inflection point. Abbreviation are explained in Table 2 and the models in Table 3. Superimposed on the overlap data (grey filled dots) in the last column is the corresponding GAM prediction (plain line).

The kittiwake diet varied over time, with *O* ranging from 16% to 88% of overlap with the previous year (60±23(SD), Fig. 2). The diet changes can be explained by the generally positive relationship with the sea temperature (ST, over 3.9°C) and with the capelin biomass (log transformed, until ca 2.4 10^6^ t) (Table 3, Fig. 2). With increasing ST and increase of capelin abundance the diet became more similar.

The guillemot diet also varied over time (16–92%, 60±23(SD)). The diet overlap decreased with time (Fig. 2). This trend taken into account, the year-to-year change in diet can be explained by the changes in the ST (Fig. 2). With increasing ST the diet became more similar until ca 4.1°C, when the relationship was reversed, i.e., the diet was more variable in extreme temperatures. With the increase of capelin abundance the diet became to some extent more variable.

The Minke whale diet was relatively stable (42–79%, 65±13(SD)). The change in diet can be explained by the changing herring abundances combined to the changes in the winter NAO index (wNAO, Fig. 2). The more abundant the herring (up to an abundance of ca 1.43 10^6^ t) and lower the wNAO the smaller was the diet overlap (Fig. 2). Hence, the diet varied more in years with high herring abundances, and in years of positive wNAO.

The cod diet was remarkably constant with very small variation compared to the other species during the studied period (56–92%, 80±10(SD)) and showed a clear positive time trend. The year-to-year fluctuations in diet can be explained by the annual variation in capelin abundance (log transformed, Fig. 2). With increasing capelin abundance the diet became less similar until ca 0.78 10^6^ t when the relationship was reversed (Fig. 2).

### Interspecific year to year variation in diet overlap

Among all pairwise comparison of predators, only two exhibited significant diet overlap, but it all cases diet overlap varied annually.

**Figure 3:**
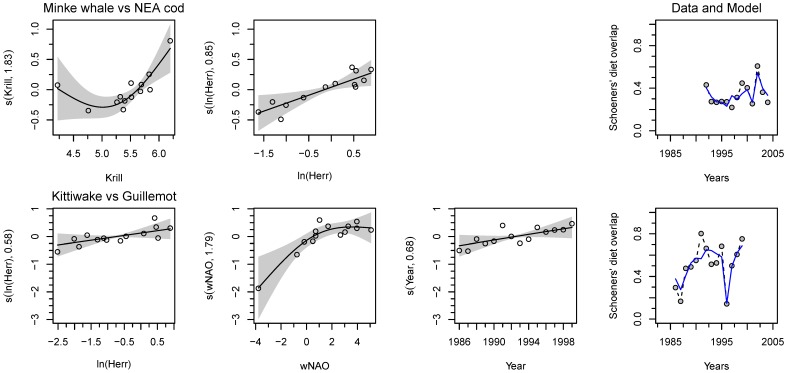
Interspecific diet overlap for the main predator species in the Barents Sea. The generalized additive models (GAMs) are presented for each pair or predator. For each plot, the x-axes show the covariate and the y-axes the partial effect that each covariate has on the response variable. The line is the smooth term effect of the considered covariate on the elasticity with the pointwise 95% confidence interval around the mean prediction (grey-shaded area). The dots are the partial residuals calculated by adding to the effect of the concerned covariate to the residuals, the model prediction at any given point is given by the sum of all partial effects plus a constant. When it applies, the dotted line locates the inflection point. Abbreviations are explained in Table 2 and the models in Table 3. Superimposed on the overlap data (grey filled dots) in the last column is the corresponding GAM prediction (plain line).

The Minke whale/cod pair had a diet overlap ranging from 22–61% (34±10(SD), Fig. 3). The change in the diet overlap between the two predators can be explained by the positive effects of herring abundance (log transformed) and of the krill abundance (Fig. 3).

The kittiwake/guillemot pair had a diet overlap ranging from 14–80% (51±20(SD), Fig. 3). The diet overlap exhibited a slight positive time trend (Fig. 3). The change in the diet overlap between the two predators can be explained by the positive effects of herring abundance (log transformed) and of the wNAO (Fig. 3). With increasing wNAO and herring abundance the diets became more similar.

The cod and the kittiwake pair (6–81%, 33±21(SD)), the Minke whale and the guillemot pair (5–77%, 33±27(SD)), the Minke whale and the kittiwake pair (11–72%, 44±24(SD)), the cod and the guillemot pair (5–54%, 31±17(SD)) had no significant diet overlap. Note that the two last pairs had near significant diet overlap.

## Discussion

Diet overlaps were obtained by using the commonly accepted Schoener's Index for niche overlap computed on stomach contents. Linton et al. [47] showed that the Schoener's index gives a more accurate representation of true overlap when the overlap is ranging between 7–90% as is the case in our study when compared to other often used indices [48]. It results that our models displayed the trend in diet overlap fairly well; their relative stiffness being likely due to the small amount of covariates used.

However, there is limitation to the diet overlap techniques. The first is the availability of the data that requires heavy logistics to obtain. This is well illustrated by the harp seal case where the data series available to us were too short for our analysis. On the same level is the spatial coverage of the data. For instance while still possible, the calculation of diet overlap index has meaning only if the two predators compared feed in the same area at the same time. This is why we have restricted the NEA cod and the Minke whale data to the southern Barents Sea when comparing with the seabirds data. To some extent there is also a similar problem with the season when the data are collected, explaining why we have also restricted the seasonal extent of the NEA cod data (Table 1). Optimally, data should be collected for all predators studied at the same geographical area, during the same season and over a sufficient amount of consecutive years.

### 1. Annual change in the diet of the main predators in the Barents Sea

Our results revealed that cod and Minke whale, the predators with a large niche breadth due to predation on a wide variety of prey species (Fig. 1 and Table S2 in File S1), had more stable diets across time than the seabirds foraging on fewer prey species (Fig. 2). In cod and Minke whale, fluctuations in prey abundances resulted in relatively small changes in use of many alternative prey species, compared to the larger changes in use of fewer prey species in the seabirds (Fig. 2). Being central place foragers during reproduction, the seabirds choice of prey is limited to the vicinity of the breeding site. It is then the local distribution of prey that explains the variation in the seabirds diet much more than the prey abundance. In this respect, the diets of cod and Minke whale appear to be more robust to fluctuations in the prey base. Indeed, wider distributions, and no spatial limitation to areas neighbouring a central place (colony) during foraging is likely important factors increasing the dietary flexibility and robustness of the cod and the Minke whale in comparison to the seabirds (e.g., large impact of prey availability on survival explaining seabird population decline such as the one observed for common guillemot in 1986–1987 [49], [50]). Nevertheless, cod, Minke whale and seabirds were negatively impacted by past fluctuations in prey abundance [36], [51], [52].

Capelin abundance is the major driver causing changes in cod and seabird diets. Nevertheless, the dietary response (i.e., change of *O*-index) to changing capelin abundance differed from U-shaped, positive and negative for cod, kittiwake and guillemot, respectively (Fig. 2). While the major prey eaten by cod is the capelin, which has highly variable abundance and distribution [33], the diet of the cod remains remarkably constant. However, the small changes observed between years are explained by the variation in capelin abundance. The U-shaped dietary response relative to capelin abundance indicates that the cod diet is similar in periods with either low or high capelin abundance, but varies during transitions between high and low capelin abundances. We suggest that the U-shape of the relationship is due to the particular dynamics of the capelin in the Barents Sea with regular periods with low abundance [26]. In such years, the cod shifts to juvenile cod and haddock as alternative prey [12], [53] or other prey with high abundance, and back to capelin when capelin stock recovers. However, it seems that juvenile cod was an important prey for adult cod only during the mid 90's capelin collapse when there was strong recruiting year classes of cod [53], but not so much during the capelin collapses in mid 80s or 2000 (Fig. S1 in File S1). Note that in the recent years, cod appears to respond to the warming by expanding its distribution range [25].

The two seabird species show remarkably mirror responses to changing capelin abundance and sea temperature. Since it was shown that the kittiwake is a competitor to the common guillemot [20], the mirror response may reflect that what is good for kittiwake is bad for the guillemot hence the remarkably similar inflection point in sea temperature at ca 4°C for both species. The change in their diet is explained by climatic variables such as sea temperature, that may be a proxy of the local condition in term of prey availability spectrum. Note that the overlap of diet between the two seabird species is stronger when winter NAO index is high which corresponds to high temperatures in the Barents Sea. This may indicate that high winter NAO index is stabilizing the prey availability around the breeding site by for example favouring one prey species over the others. A study on the spatial distribution showed that in the Barents Sea the seabird distribution at sea was relatively stable over the years (Fauchald pers. comm.).

Similarly to the cod, the Minke whale displays a relatively constant diet. The small changes in its diet are explained by variation in the abundance of the juvenile herring in the Barents Sea and not of the capelin. However, the Minke whale's body condition was found to be poorer in years when both capelin and herring was at a low abundance level [36] indicating a dependence on capelin availability. The diet was also more similar in years with low herring abundance. Years with little herring in the diet coincided with periods with little capelin but increased krill in the diet (Fig. S1 in File S1); krill is an important alternative prey for these whales when pelagic fish abundances are low [36]. However, during the recent years, the Minke whale distribution was relatively constant and independent of prey distribution [54], similarly to the seabirds (Fauchald pers. comm.). The past decade showed an increasing abundances of krill and shrimp associated with large stocks of demersal and pelagic fish in the Barents Sea [7]. During this recent period the whale condition might have remain good despite the period of low capelin abundance thanks to alternate prey (e.g. krill). Unfortunately our data on Minke whale do not cover the recent years; i.e., they stop in 2004. However, our model may have caught this effect through the positive effect of winter NAO index on the diet similarity; positive NAO being the signature of the later years (Fig S2 in File S1).

### 2. Annual change in diet overlap of the major predators in the Barents Sea

When exploring the trophic interaction between predators we should always keep in mind that the species are not representing the same biomass in carbon. For instance in the Barents Sea, the cod biomass is some hundred mg C m^−2^, the whales ca 100 mg C m^−2^, while the seabirds all together are only up to 2.5 mg C m^−2^ [55]. This difference in biomass must be taken in to account when comparing interspecific diets. For instance, a diet overlap between cod and kittiwake may indicate a potential competition of cod on kittiwake but not the reverse (or very locally). On the other hand, Minke whale and cod populations or the two seabird populations having similarly scaled biomass may engage in a direct two-way competition [20]. Another factor to consider is that digestion rate can be different between species – from 3–10 hours needed for full digestion of fish (sandeel and whiting) in seabirds [56] and up to 1–3 days in cod (capelin, herring, shrimp and other prey) (see refs in [16]). More important is that the digestion rates ratio between prey type (e.g., digestion rate for crustacean/digestion rate for fish prey…) is similar for the predators compared. If not, some prey species may be overly represented in the diet of some predators and not others. Unfortunately such information is not available. Among the pairs tested only the Minke whale/NEA cod and kittiwake/common guillemot pairs display significant diet overlap (i.e., have a regular diet overlap over the years, Fig. 3). Food competition may thus occur between Minke whale and cod, but the implications for interspecific competition need to be mathematically tested [20]. Taking into account the difference in biomass and well known effect of cod predation on capelin [26] we may also find food competition of cod on seabirds, notably with kittiwake where the overlap is nearly significant.

Changes in pairs of diet overlap are explained by a positive effect of herring and/or krill abundance. Herring is an essential food source, e.g., during chick raising at Kharlov (Krasnov pers. com.), that may explain why the two seabird populations have a more similar diet when the juvenile herring are abundant in the Barents Sea. The same is true for the Minke whale and the cod, however, previous works have shown that both Minke whale [36] and cod [57] switch to krill and amphipods as prey in periods with low herring and capelin abundance. It seems that there is two alternative states where cod and Minke whale have high similarity in their diet; one with high abundance of capelin/herring (the two stocks show a similar dynamic with time, see Fig S2 in File S1) and one with low abundance of these pelagic prey but high abundance of krill (note that krill and capelin populations tend to have inverse temporal dynamic, Fig. S2 in File S1).

### 3. Conclusions

The Barents Sea predators demonstrated a diversity both in their diets, and in change in diets within and between species. Also the responses to possible drivers of diet change, such as abundances of key prey species and ocean climate were diverse, both within species and between pairs of species. The potential for interspecific competition could perhaps be strongest if top predator diets became more similar when prey abundances were low, i.e. that the top predators were switching to the same alternative prey species. However, the dietary response diversity observed in this study indicate that the top predator community could be relatively robust to changes in the ecosystem. As with the diversity of species that contribute to the same ecosystem function is regarded as an important property for ecosystem resilience [23], [58], [59], the diversity of responses [23] to environmental changes within functional groups will increase the probability of compensation for one species by the others and thereby secure the continuation of an ecosystem function [60].

## Supporting Information

File S1 **Figure S1. Diet of the main predator species in the Barents Sea over time.** Note that for the black-legged kittiwakes and common guillemots the amphipods and krill prey species where not dissociated and are assembled in one category “krill”. There are two minke whale diet plots: for the whole Barents Sea (left) and restricted to the Southern Barents Sea part (70–74°N and 20–40°E). The first data are used to analyse the change in the minke whale diet over time and to compare with the diet of the NEA cod. The second data are used to compare with the diet of the seabirds that are central place foragers and limited to the Southern Barents Sea during reproduction (period when the seabird diet data were collected). There are three NEA cod plots: for the ICES data (1984–2009) used for the intraspecific analysis and for restricted area of the Barents Sea to compare with the seabirds' diet (March to July, 68–72°N and 20–40°E) and with the minke whale's diet (July to September, 70–80°N and 5–40°E). **Figure S2. Time series used as explanatory variables in the study.** Data for the winter NAO come from https://climatedataguide.ucar.edu/sites/default/files/climate_index_files/nao_station_djfm.txt. Data for the sea temperature come from PINRO. They are yearly average sea temperature measured monthly at 0–200 m depth on the Russian Kola meridian transect (33° 30′ E, 70° 30′ N to 72° 30′ N). Data for capelin and herring biomass come from ICES report (Table 9.5 p 498 in ICES 2012). **Figure S3. Interspecific diet overlap for the main predator species in the Barents Sea.** Change of diet from one year to another is presented by a Schoeners' diet overlap index (grey filled dots). Higher is the index higher is the overlap. **Table S1.** Diet of the different predators. **Table S2.** Prey species and categories used for the calculation of the Schoeners' index.
(DOCX)
