# Supplementary material for: Temporal Dynamics of Top Predators Interactions in the Barents Sea
Source: PLoS One. 2014 Nov 3;9(11):e110933. doi: 10.1371/journal.pone.0110933 (PMC4218717; doi:10.1371/journal.pone.0110933)
Supplement: File S1 — Figure S1. Diet of the main predator species in the Barents Sea over time. Note that for the black-legged kittiwakes and common guillemots the amphipods and krill prey species where not dissociated and are assembled in one category “krill”. There are two minke whale diet plots: for the whole Barents Sea (left) and restricted to the Southern Barents Sea part (70–74°N and 20–40°E). The first data are used to analyse the change in the minke whale diet over time and to compare with the diet of the NEA cod. The second data are used to compare with the diet of the seabirds that are central place foragers and limited to the Southern Barents Sea during reproduction (period when the seabird diet data were collected). There are three NEA cod plots: for the ICES data (1984–2009) used for the intraspecific analysis and for restricted area of the Barents Sea to compare with the seabirds' diet (March to July, 68–72°N and 20–40°E) and with the minke whale's diet (July to September, 70–80°N and 5–40°E). Figure S2. Time series used as explanatory variables in the study. Data for the winter NAO come from https://climatedataguide.ucar.edu/sites/default/files/climate_index_files/nao_station_djfm.txt. Data for the sea temperature come from PINRO. They are yearly average sea temperature measured monthly at 0–200 m depth on the Russian Kola meridian transect (33° 30′ E, 70° 30′ N to 72° 30′ N). Data for capelin and herring biomass come from ICES report (Table 9.5 p 498 in ICES 2012). Figure S3. Interspecific diet overlap for the main predator species in the Barents Sea. Change of diet from one year to another is presented by a Schoeners' diet overlap index (grey filled dots). Higher is the index higher is the overlap. Table S1. Diet of the different predators. Table S2. Prey species and categories used for the calculation of the Schoeners' index. (DOCX) [file pone.0110933.s001.docx]

**Supporting information**

**Temporal Dynamics of Top Predators Interactions in the Barents Sea**

Joël M. Durant, Mette Skern-Mauritzen, Yuri V. Krasnov, Natalia G. Nikolaeva, Ulf Lindstrøm, Andrey Dolgov

**Table S1. Diet of the different predators.**

|  | **Black-legged kittiwake** | **Common guillemot** | **Minke whale** | **Northeast Arctic cod** |
| --- | --- | --- | --- | --- |
|  | *Rissa tridactyla* | *Uria aalge* | *Balaenoptera acutorostrata* | *Gadus morhua* |
| Years | 1982-1999 | 1984-1999 | 1992-2004 | 1984-2009 |
| n | 18 | 16 | 13 | 26 |
| Latitude | 68°78 | 68°78 | 69°83-80°28 | Barents Sea |
| Longitude | 37°33 | 37°33 | 6°54-38°65 |  |
| Niche breadth D | 1.09 ± 0.28(SD) | 0.79 ± 0.31(SD) | 1.25 ± 0.21(SD) | 1.51 ± 0.21(SD) |
| (range) | (0.45 – 1.65) | (0.39 – 1.42) | (0.66 – 1.49) | (1.00 – 1.81) |
| Prey species | Herring | Herring | Herring | Herring |
|  | Capelin | Capelin | Capelin | Capelin |
|  | Cod | Cod | Cod | Cod |
|  | Haddock | Sandeel | Haddock | Haddock |
|  | Sandeel | Redfish | Saithe | Blue whiting |
|  | Salmon | Wolfish | Sandeel | Polar cod |
|  | Bullhead | Blenny | Mackerel | Redfish |
|  | Butterfish |  | Krill | Long rough dab |
|  | Blenny |  | Unidentified codfish | Greenland halibut |
|  | Bottom fish |  | Other fish | Krill |
|  | Other fish |  |  | Amphipods |
|  | Crustaceans |  |  | Shrimp |
|  | Offal |  |  | Other |

D is the Shannon-Wiener niche breadth index calculated as follows:

D = - Σ P_i_*ln(P_i_)

where p_i_ is the proportion of the species considered feeding on prey species/group i.

**Table S2. Prey species and categories used for the calculation of the Schoeners’ index.**

| **Category** | **species** | |
| --- | --- | --- |
| Capelin | *Mallotus villosus* | |
| Herring | *Clupea harengus* | |
| Cod | *Gadus morhua* | |
| Haddock | *Melanogrammus aeglefinus* | |
| Other gadoids | e.g., Blue whiting (*Micromesistius* *poutassou*) or unidentified gadoids | |
| Saithe | *Pollachius* *virens* | |
| Polar cod | *Boreogadus* *saida* | |
| Sandeel | Ammodytidae | |
| Redfish | *Sebastes sp.* | |
| Flatfish | e.g., long rough dab (*Hippoglossoides platessoides*), Greenland halibut (*Reinhardtius hippoglossoides*) etc | |
| Krill | Euphausiids | |
| Amphipods | Hyperiids | |
| Crustaceans | e.g., shrimp *Pandalus borealis* | |
| Other fishes | every fish not in the above categories | |
| Offal | fish rest |  |
| Other | stomach content of cod: mush | |

To calculate the diet overlap between species we needed the same number of categories for each predator diet. We have put in the same categories some prey such as halibut and plaice in the flatfish category... Intraspecific diet overlap analysis was conducted on unmodified data as extract from our sources (see Table S1).

**Figure S1. Diet of the main predator species in the Barents Sea over time**.

Note that for the black-legged kittiwakes and common guillemots the amphipods and krill prey species where not dissociated and are assembled in one category “krill”. There are two minke whale diet plots: for the whole Barents Sea (left) and restricted to the Southern Barents Sea part (70-74°N and 20-40°E). The first data are used to analyse the change in the minke whale diet over time and to compare with the diet of the NEA cod. The second data are used to compare with the diet of the seabirds that are central place foragers and limited to the Southern Barents Sea during reproduction (period when the seabird diet data were collected). There are three NEA cod plots: for the ICES data (1984-2009) used for the intraspecific analysis and for restricted area of the Barents Sea to compare with the seabirds’ diet (March to July, 68-72°N and 20-40°E ) and with the minke whale’s diet (July to September, 70-80°N and 5-40°E).

**Figure S2. Time series used as explanatory variables in the study**.

Data for the winder NAO come from https://climatedataguide.ucar.edu/sites/default/files/climate_index_files/nao_station_djfm.txt.

Data for the sea temperature come from PINRO. They are yearly average sea temperature measured monthly at 0-200 m depth on the Russian Kola meridian transect (33° 30’ E, 70° 30’ N to 72° 30’ N).

Data for capelin and herring biomass come from ICES report (Table 9.5 p 498 in ICES 2012).

**Figure S3**. **Interspecific diet overlap for the main predator species in the Barents Sea**.

Change of diet from one year to another is presented by a Schoeners’ diet overlap index (grey filled dots). Higher is the index higher is the overlap.
